# Supplementary material for: The PCNA unloader Elg1 promotes recombination at collapsed replication forks in fission yeast
Source: eLife. 2019 May 31;8:e47277. doi: 10.7554/eLife.47277 (PMC6544435; doi:10.7554/eLife.47277)
Supplement: Supplementary file 1. [file elife-47277-supp1.docx]

**Supplementary File 1:** Direct repeat recombinant frequencies

| Genotype and strain number | *RTS1* orientation | Position of direct repeat | Number of colonies analysed | Ade^+^ His^+^  recombinant  frequency (x 10^-4^)^a^ | | Ade^+^ His^-^  recombinant  frequency (x 10^-4^)^a^ | |
| --- | --- | --- | --- | --- | --- | --- | --- |
|  |  |  |  | Mean | *P* value^b^ | Mean | *P* value^b^ |
| wild-type  MCW4712 | IO | Flanking *RTS1* (0 kb site) | 26 | 1.31  (+/- 0.79) | - | 3.33  (+/- 0.93) | - |
| *elg1*∆  MCW7706 | IO | Flanking *RTS1* (0 kb site) | 26 | 2.08  (+/- 0.55) | <0.0001^c^ | 4.48  (+/- 1.05) | <0.0001^c^ |
| wild-type  MCW4713 | AO | Flanking *RTS1* (0 kb site) | 25 | 147.5  (+/- 51.3) | <0.0001^c^ | 103.4  (+/- 55.8) | <0.0001^c^ |
| *elg1*∆  MCW7708 | AO | Flanking *RTS1* (0 kb site) | 26 | 7.32  (+/- 1.50) | <0.0001^d^ | 28.0  (+/- 8.01) | <0.0001^d^ |
| wild-type  MCW7259 | AO | 12.4 kb downstream of *RTS1* | 14 | 3.92  (+/- 2.27) | - | 80.1  (+/- 18.9) | - |
| *elg1*∆  MCW8191 | AO | 12.4 kb downstream of *RTS1* | 18 | 0.85  (+/- 0.73) | <0.0001^e^ | 13.62  (+/- 6.78) | <0.0001^e^ |
| *ori-1253*∆  MCW7295 | AO | 12.4 kb downstream of *RTS1* | 16 | 83.6  (+/- 34.0) | <0.0001^e^ | 1046.7  (+/- 468.9) | <0.0001^e^ |
| *ori-1253*∆  *elg1*∆  MCW8290 | AO | 12.4 kb downstream of *RTS1* | 16 | 5.30  (+/- 3.65) | <0.0001^f^ | 93.6  (+/- 16.5) | <0.0001^f^ |
| *pcn1^+^*  MCW9394 | IO | Flanking *RTS1* (0 kb site) | 26 | 4.04  (+/- 2.01) | <0.0001^c^ | 6.32  (+/- 3.60) | <0.0001^c^ |
| *pcn1^+^ elg1*∆  MCW9390 | IO | Flanking *RTS1* (0 kb site) | 16 | 3.67  (+/- 1.90) | 0.298^g^ | 7.75  (+/- 4.77) | 0.231^g^ |
| *pcn1^D150E^*  MCW9183 | IO | Flanking *RTS1* (0 kb site) | 21 | 8.88  (+/- 3.01) | <0.0001^g^ | 17.7  (+/- 8.59) | <0.0001^g^ |
| *pcn1^D150E^ elg1*∆  MCW9187 | IO | Flanking *RTS1* (0 kb site) | 23 | 11.1  (+/- 2.74) | 0.009^h^ | 18.2  (+/- 3.96) | 0.658^h^ |
| *pcn1^+^*  MCW9396 | AO | Flanking *RTS1* (0 kb site) | 26 | 144.9  (+/- 66.7) | <0.0001^g^ | 135.0  (+/- 65.9) | <0.0001^g^ |
| *pcn1^+^ elg1*∆  MCW9392 | AO | Flanking *RTS1* (0 kb site) | 25 | 16.5  (+/- 11.2) | <0.0001^i^ | 49.3  (+/- 28.2) | <0.0001^i^ |
| *pcn1^D150E^*  MCW9185 | AO | Flanking *RTS1* (0 kb site) | 20 | 157.7  (+/- 86.5) | 0.669^i^ | 114.5  (+/- 52.0) | 0.361^i^ |
| *pcn1^D150E^ elg1*∆  MCW9189 | AO | Flanking *RTS1* (0 kb site) | 22 | 122.5  (+/- 47.7) | 0.197^j^ | 111.8  (+/- 34.3) | 0.871^j^ |
| *srs2*∆  FO1750 | AO | Flanking *RTS1* (0 kb site) | 20 | 1046.5  (+/- 183.7) | <0.0001^d^ | 467.9  (+/- 175.7) | <0.0001^d^ |
| *srs2*∆ *elg1*∆  MCW8330 | AO | Flanking *RTS1* (0 kb site) | 20 | 81.7  (+/- 13.7) | <0.0001^k^ | 106.3  (+/- 30.2) | <0.0001^k^ |
| *fbh1*∆  FO1816 | AO | Flanking *RTS1* (0 kb site) | 17 | 723.2  (+/- 194.9) | <0.0001^d^ | 2093.6  (+/- 678.1) | <0.0001^d^ |
| *fbh1*∆ *elg1*∆  MCW8946 | AO | Flanking *RTS1* (0 kb site) | 15 | 394.3  (+/- 192.8) | <0.0001^l^ | 2181.3  (+/- 1026) | 0.710^l^ |
| wild-type  MCW8023 | AO | Flanking *RTS1* (0 kb site) + 5 kb spacer | 18 | 213.2  (+/- 107.7) | - | 1038.1  (+/- 424.6) | - |
| *elg1*∆  MCW8941 | AO | Flanking *RTS1* (0 kb site) + 5 kb spacer | 18 | 11.1  (+/- 8.33) | <0.0001^m^ | 87.7  (+/- 39.6) | <0.0001^m^ |
| *rad51*∆  MCW8136 | AO | Flanking *RTS1* (0 kb site) + 5 kb spacer | 15 | 18.4  (+/- 24.8) | <0.0001^m^ | 963.7  (+/- 433.4) | 0.605^m^ |
| *rad51*∆ *elg1*∆  MCW8943 | AO | Flanking *RTS1* (0 kb site) + 5 kb spacer | 16 | 0.67  (+/- 1.97) | <0.0001^n^ | 514.4  (+/- 383.2) | <0.001^n^ |

^a^ The values in parentheses are the standard deviations about the mean.

^b^ *P* values are derived from the Mann-Whitney U test comparing the mean values as indicated.

^c^ Compared to the equivalent mean recombinant frequency in MCW4712.

^d^ Compared to the equivalent mean recombinant frequency in MCW4713.

^e^ Compared to the equivalent mean recombinant frequency in MCW7259.

^f^ Compared to the equivalent mean recombinant frequency in MCW7295 and MCW8191.

^g^ Compared to the equivalent mean recombinant frequency in MCW9394.

^h^ Compared to the equivalent mean recombinant frequency in MCW9183.

^i^ Compared to the equivalent mean recombinant frequency in MCW9396.

^j^ Compared to the equivalent mean recombinant frequency in MCW9185.

^k^ Compared to the equivalent mean recombinant frequency in FO1750.

^l^ Compared to the equivalent mean recombinant frequency in FO1816.

^m^ Compared to the equivalent mean recombinant frequency in MCW8023.

^n^ Compared to the equivalent mean recombinant frequency in MCW8136.
