# Supplementary material for: The PCNA unloader Elg1 promotes recombination at collapsed replication forks in fission yeast
Source: eLife. 2019 May 31;8:e47277. doi: 10.7554/eLife.47277 (PMC6544435; doi:10.7554/eLife.47277)
Supplement: Supplementary file 2. [file elife-47277-supp2.docx]

**Supplementary File 2:** List of *S. pombe* strains used in this study (in order of appearance)

| Strain | Mating type | Genotype | Source |
| --- | --- | --- | --- |
| MCW4712 | *h^+^* | *ade6-M375 int*::*pUC8/his3^+^/RTS1-IO/ade6-L469 ura4-D18 his3-D1 leu1-32 arg3-D4* | (Ahn et al., 2005) |
| MCW7706 | *h^+^* | *elg1*∆::*natMX6^1^ ade6-M375 int*::*pUC8/his3^+^/RTS1-IO/ade6-L469 ura4-D18 his3-D1 leu1-32 arg3-D4* | This study |
| MCW4713 | *h^+^* | *ade6-M375 int*::*pUC8/his3^+^/RTS1-AO/ade6-L469 ura4-D18 his3-D1 leu1-32 arg3-D4* | (Ahn et al., 2005) |
| MCW7708 | *h^+^* | *elg1*∆::*natMX6 ade6-M375 int*::*pUC8/his3^+^/RTS1-AO/ade6-L469 ura4-D18 his3-D1 leu1-32 arg3-D4* | This study |
| MCW7259 | *h^+^* | *ade6∆*::*RTS1-AO-hphMX4* (12.4kb from *ade6*)int::*ade6-M375/pUC8/his3^+^/ade6-L469/kanMX6 ura4-D18 his3-D1 leu1-32 arg3-D4* | (Nguyen et al., 2015) |
| MCW8191 | *h^+^* | *elg1*∆::*natMX6 ade6∆*::*RTS1-AO-hphMX4* (12.4kb from *ade6*)int::*ade6-M375/pUC8/his3^+^/ade6-L469/kanMX6 ura4-D18 his3-D1 leu1-32 arg3-D4* | This study |
| MCW7295 | *h^+^* | *oriIII-1253∆*::*natMX4 ade6∆*::*RTS1-AO-hphMX4* (12.4kb from *ade6*)int::*ade6-M375/pUC8/his3^+^/ade6-L469/kanMX6 ura4-D18 his3-D1 leu1-32 arg3-D4* | (Nguyen et al., 2015) |
| MCW8290 | *h^+^* | *elg1*∆::*natMX6 oriIII-1253∆*::*arg3^+^ ade6∆*::*RTS1-AO-hphMX4* (12.4kb from *ade6*)int::*ade6-M375/pUC8/his3^+^/ade6-L469/kanMX6 ura4-D18 his3-D1 leu1-32 arg3-D4* | This study |
| MCW7065 | *h^+^* | *ade6-M375 int*::*pUC8/lacO^115^/his3^+^/RTS1-AO/ade6-L469 lys1^-^*::*Pnmt41-NLS-lacI-tdKatushka2-hphMX4 rad52^+^*::*YFP-kanMX6 ura4^-^*::*pECFP-PCNA^+^ his3-D1 leu1-32 arg3-D4* | (Nguyen et al., 2015) |
| MCW7965 | *h^+^* | *elg1*∆::*natMX6 ade6-M375 int*::*pUC8/lacO^115^/his3^+^/RTS1-AO/ade6-L469 lys1^-^*::*Pnmt41-NLS-lacI-tdKatushka2-hphMX4 rad52^+^*::*YFP-kanMX6 ura4^-^*::*pECFP-PCNA^+^ his3-D1 leu1-32 arg3-D4* | This study |
| MCW9394 | *h^+^* | *pcn1^+^*::*natMX4 ade6-M375 int*::*pUC8/his3^+^/RTS1-IO/ade6-L469 ura4-D18 his3-D1 leu1-32 arg3-D4* | This study |
| MCW9390 | *h^+^* | *elg1*∆::*kanMX6^2^ pcn1^+^*::*natMX4 ade6-M375 int*::*pUC8/his3^+^/RTS1-IO/ade6-L469 ura4-D18 his3-D1 leu1-32 arg3-D4* | This study |
| MCW9183 | *h^?^* | *pcn1^D150E^*::*natMX4 ade6-M375 int*::*pUC8/his3^+^/RTS1-IO/ade6-L469 ura4-D18 his3-D1 leu1-32 arg3-D4* | This study |
| MCW9187 | *h^?^* | *elg1*∆::*kanMX6 pcn1^D150E^*::*natMX4 ade6-M375 int*::*pUC8/his3^+^/RTS1-IO/ade6-L469 ura4-D18 his3-D1 leu1-32 arg3-D4* | This study |
| MCW9396 | *h^+^* | *pcn1^+^*::*natMX4 ade6-M375 int*::*pUC8/his3^+^/RTS1-AO/ade6-L469 ura4-D18 his3-D1 leu1-32 arg3-D4* | This study |
| MCW9392 | *h^+^* | *elg1*∆::*kanMX6 pcn1^+^*::*natMX4 ade6-M375 int*::*pUC8/his3^+^/RTS1-AO/ade6-L469 ura4-D18 his3-D1 leu1-32 arg3-D4* | This study |
| MCW9185 | *h^?^* | *pcn1^D150E^*::*natMX4 ade6-M375 int*::*pUC8/his3^+^/RTS1-AO/ade6-L469 ura4-D18 his3-D1 leu1-32 arg3-D4* | This study |
| MCW9189 | *h^?^* | *elg1*∆::*kanMX6 pcn1^D150E^*::*natMX4 ade6-M375 int*::*pUC8/his3^+^/RTS1-AO/ade6-L469 ura4-D18 his3-D1 leu1-32 arg3-D4* | This study |
| FO1750 | *h^+^* | *srs2*∆::*ura4^+^ ade6-M375 int*::*pUC8/his3^+^/RTS1-AO/ade6-L469 ura4-D18 his3-D1 leu1-32 arg3-D4* | (Lorenz et al., 2009) |
| MCW8330 | *h^+^* | *elg1*∆::*natMX6 srs2*∆::*ura4^+^ ade6-M375 int*::*pUC8/his3^+^/RTS1-AO/ade6-L469 ura4-D18 his3-D1 leu1-32 arg3-D4* | This study |
| MCW1221 | *h^+^* | *ura4-D18 his3-D1 leu1-32 arg3-D4* | (Lorenz et al., 2009) |
| MCW7586 | *h^+^* | *elg1*∆::*natMX6 ura4-D18 his3-D1 leu1-32 arg3-D4* | This study |
| MCW1017 | *h^+^* | *srs2*∆::*ura4^+^ ura4-D18 his3-D1 leu1-32 arg3-D4* | (Lorenz et al., 2009) |
| MCW8332 | *h^+^* | *elg1*∆::*natMX6 srs2*∆::*ura4^+^ ura4-D18 his3-D1 leu1-32 arg3-D4* | This study |
| FO1816 | *h^+^* | *fbh1*∆::*kanMX6 ade6-M375 int*::*pUC8/his3^+^/RTS1-AO/ade6-L469 ura4-D18 his3-D1 leu1-32 arg3-D4* | (Lorenz et al., 2009) |
| MCW8946 | *h^-^* | *elg1*∆::*natMX6 fbh1*∆::*kanMX6 ade6-M375 int*::*pUC8/his3^+^/RTS1-AO/ade6-L469 ura4-D18 his3-D1 leu1-32 arg3-D4* | This study |
| MCW7638 | *h^+^* | *ade6-M375 int*::*pUC8/lacO^115^/his3^+^/RTS1-AO/ade6-L469 lys1^-^*::*Pnmt41-NLS-lacI-tdKatushka2-hphMX4 rad52^+^*::*YFP-kanMX6 rad51^+^*::*ECFP-rad51^+^-arg3^+^ ura4-D18*::*rad51^+^-ura4^+^ his3-D1 leu1-32 arg3-D4* | (Nguyen et al., 2015) |
| MCW8921 | *h^-^* | *elg1*∆::*natMX6 ade6-M375 int*::*pUC8/lacO^115^/his3^+^/RTS1-AO/ade6-L469 lys1^-^*::*Pnmt41-NLS-lacI-tdKatushka2-hphMX4 rad52^+^*::*YFP-kanMX6 rad51^+^*::*ECFP-rad51^+^-arg3^+^ ura4-D18*::*rad51^+^-ura4^+^ his3-D1 leu1-32 arg3-D4* | This study |
| MCW8023 | *h^+^* | *ade6-M375 int*::*pUC8/5000 bp spacer/his3^+^/RTS1-AO/ade6-L469 ura4-D18 his3-D1 leu1-32 arg3-D4* | (Morrow et al., 2017) |
| MCW8941 | *h^+^* | *elg1*∆::*natMX6 ade6-M375 int*::*pUC8/5000 bp spacer/his3^+^/RTS1-AO/ade6-L469 ura4-D18 his3-D1 leu1-32 arg3-D4* | This study |
| MCW8136 | *h^+^* | *rad51*∆*::arg3^+^ ade6-M375 int*::*pUC8/5000 bp spacer/his3^+^/RTS1-AO/ade6-L469 ura4-D18 his3-D1 leu1-32 arg3-D4* | (Morrow et al., 2017) |
| MCW8943 | *h^+^* | *elg1*∆::*natMX6 rad51*∆*::arg3^+^ ade6-M375 int*::*pUC8/5000 bp spacer/his3^+^/RTS1-AO/ade6-L469 ura4-D18 his3-D1 leu1-32 arg3-D4* | This study |

^1^ *elg1*∆::*natMX6* was derived from Sp457, which was a gift from Stuart MacNeill

^2^ *elg1*∆::*kanMX6* was derived from Sp373, which was a gift from Stuart MacNeill
